# Planar and van der Waals heterostructures for vertical tunnelling single electron transistors

**DOI:** 10.1038/s41467-018-08227-1

**Published:** 2019-01-16

**Authors:** Gwangwoo Kim, Sung-Soo Kim, Jonghyuk Jeon, Seong In Yoon, Seokmo Hong, Young Jin Cho, Abhishek Misra, Servet Ozdemir, Jun Yin, Davit Ghazaryan, Matthew Holwill, Artem Mishchenko, Daria V. Andreeva, Yong-Jin Kim, Hu Young Jeong, A-Rang Jang, Hyun-Jong Chung, Andre K. Geim, Kostya S. Novoselov, Byeong-Hyeok Sohn, Hyeon Suk Shin

**Affiliations:** 1 Department of Energy Engineering, Ulsan National Institute of Science & Technology (UNIST), Ulsan, 44919 Republic of Korea; 2 Department of Chemistry, Seoul National University, Seoul, 08826 Republic of Korea; 3 Department of Chemistry, UNIST, Ulsan, 44919 Republic of Korea; 4 Department of Physics, Konkuk University, Seoul, 05029 Republic of Korea; 5 School of Physics and Astronomy, University of Manchester, Manchester, M13 9PL United Kingdom; 6 Department of Physics, Indian Institute of Technology Madras, Chennai, 600036 India; 7 Department of Physics, National Research University Higher School of Economics, Staraya Basmannaya 21/4, Moscow, 105066 Russian Federation; 8 Department of Materials Science and Engineering, National University of Singapore, Singapore, 117575 Singapore; 9 Center for Multidimensional Carbon Materials, Institute of Basic Science (IBS), Ulsan, 44919 Republic of Korea; 10 UNIST Central Research Facilities (UCRF), UNIST, Ulsan, 44919 Republic of Korea; 11 Low Dimensional Carbon Material Center, UNIST, Ulsan, 44919 Republic of Korea; 12 Present Address: Carbon Composite Materials Research Center, Korea Institute of Science and Technology (KIST), Wanju, 55324 Republic of Korea

## Abstract

Despite a rich choice of two-dimensional materials, which exists these days, heterostructures, both vertical (van der Waals) and in-plane, offer an unprecedented control over the properties and functionalities of the resulted structures. Thus, planar heterostructures allow p-n junctions between different two-dimensional semiconductors and graphene nanoribbons with well-defined edges; and vertical heterostructures resulted in the observation of superconductivity in purely carbon-based systems and realisation of vertical tunnelling transistors. Here we demonstrate simultaneous use of in-plane and van der Waals heterostructures to build vertical single electron tunnelling transistors. We grow graphene quantum dots inside the matrix of hexagonal boron nitride, which allows a dramatic reduction of the number of localised states along the perimeter of the quantum dots. The use of hexagonal boron nitride tunnel barriers as contacts to the graphene quantum dots make our transistors reproducible and not dependent on the localised states, opening even larger flexibility when designing future devices.

## Abstract (web summary)

The possibility to combine planar and van der Waals heterostructures holds great promise for nanoscale electronic devices. Here, the authors report an innovative method to synthesise embedded graphene quantum dots within hexagonal boron nitride matrix for vertical tunnelling single electron transistor applications.

## Introduction

Graphene quantum dots (GQDs) and graphene nanowires have been attracting attention because of the linear spectrum obeyed by the quasiparticles^1,2^ (zero mass allows one to reach large quantisation energy, comparable with the room temperature, for relatively large quantum dots^3,4^), small spin-orbit interaction^5,6^, good chemical stability^7^ and the ability to support high currents. At the same time, the transport properties of such quantum dots, which are typically carved out of large sheets of graphene, are dominated by the localised edge states^1,8,9^. Furthermore, arranging tunnelling contacts (usually carved graphene constriction) to such quantum dots with the specific, reproducible conductivity is a separate challenge.

Here we use planar^10–12^ and vertical^13–15^ heterostructures to mitigate the issues with the localised states both at the edges of the quantum dots and at the edges of the contacts. We propose to form GQDs inside the hexagonal boron nitride (hBN) matrix through catalytic-assisted substitution of boron and nitrogen atoms by carbon^16^. As the lattices of graphene and hBN are very similar—most of the bonds in a GQD become properly terminated, which minimises the number of such localised states. Thus, the edges of a GQD become effectively passivated with hBN. We would like to stress that our method allows the formation of GQDs with specific size, and can be extended to other structures and devices. We then used hBN tunnelling barrier and graphene electrodes in order to form contacts to such quantum dots, creating single-electron tunnelling transistors. Such method results in very precise, reproducible contact resistance and the graphene-hBN interface free of localised states.

## Results

### Formation of GQDs

**Fig. 1:**
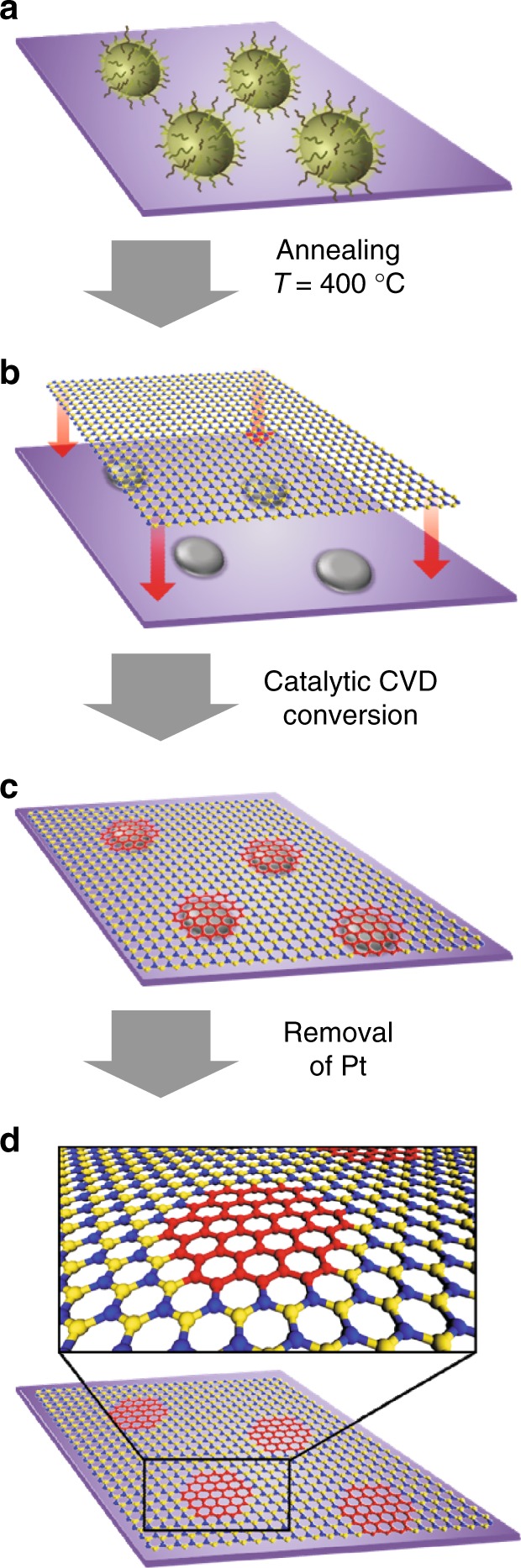
The fabrication steps of GQD-hBN in-plane heterostructure based on hBN to graphene conversion catalysed by Pt NPs. **a** The self-assembly of diblock copolymer micelles PS-P4VP with H_2_PtCl_6_ on Si/SiO_2_ substrate. **b** Transfer of hBN monolayer on SiO_2_ substrate covered by Pt NPs (blue spheres—boron atoms, yellow spheres—nitrogen). **c** Formation of the GQDs on top of an array of Pt NPs by catalytically assisted CVD (red spheres—carbon atoms). **d** The obtained in-plane GQD-hBN heterostructure after the removal of Pt NPs

**Fig. 2:**
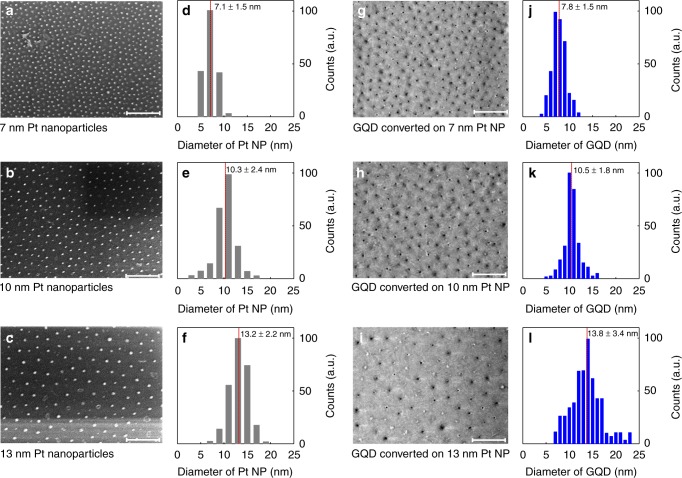
Size and spatial distribution of the GQDs. **a**–**c**, The SEM images of 7, 10 and 13 nm sized self-assembled arrays of Pt NPs on SiO_2_ substrates, respectively. Scale bar 300 nm. **d**–**f** Corresponding size distribution histograms of Pt NPs on SiO_2_ substrates. Numbers give the average (marked by red lines) and the standard deviation. **g**–**i** The SEM images of GQD-hBN in-plane heterostructures prepared on pristine SiO_2_. Scale bar 300 nm. **j**–**l** Corresponding size histograms of GQD-hBN samples. Numbers give the average (marked by red lines) and the standard deviation

In order to fabricate the in-plane graphene/hBN heterostructures, we used a conversion reaction on a patterned Pt-SiO_2_ substrate described in ref. ^16^. Based on the spatially controlled conversion, the growth of in-plane GQD-hBN heterostructure was achieved on a SiO_2_ substrate covered by an array of platinum (Pt) nanoparticles (NP), as illustrated in Fig. 1. The high-quality hBN monolayer was first grown on Pt foils via chemical vapour deposition (CVD), using ammonia borane as a precursor^17^ (the experimental details for the growth and the characterisation of monolayer hBN are provided in Methods and Supplementary Figure 1). Next, the hBN film was transferred onto an array of Pt NPs spread over SiO_2_ substrate, prepared with the aid of self-patterning diblock copolymer micelles^18^. The Pt NPs were obtained by spin-coating of a single layer of polystyrene-block-poly(4-vinylpyridine) (PS-P4VP) micelles, with an H_2_PtCl_6_ precursor for Pt NPs within their cores, followed by the annealing at 400 °C. Then, the conversion of the hBN sheet to graphene on the array of Pt NPs on the SiO_2_ substrate was accomplished at ~950 °C in methane/argon atmosphere. During the reaction, the hBN on top of Pt NPs was selectively converted to graphene, with the formation of uniform GQD arrays embedded in the hBN film (Supplementary Figure 2). Notably, depending on the molecular weight of the diblock copolymer, the size of the Pt NPs was controlled in the range of 7–13 nm. The scanning electron microscopy (SEM) images presented in Fig. 2a–c demonstrates the uniform arrays of Pt NPs with diameters of ~ 7, 10 and 13 nm (Fig. 2d–f). Next, the as-prepared GQD-hBN in-plane heterostructure was placed in aqua regia solution to remove Pt NPs (Fig. 2g–i), and finally, transferred onto arbitrary substrates for further characterisation and processing. Note, that the area of obtained GQDs is comparable to the size of the Pt NPs, as shown in Fig. 2. The removal of the Pt NPs was confirmed by X-ray photoelectron spectroscopy (XPS) and transmission electron microscopy (TEM) (Supplementary Figures 3–4)

**Fig. 3:**
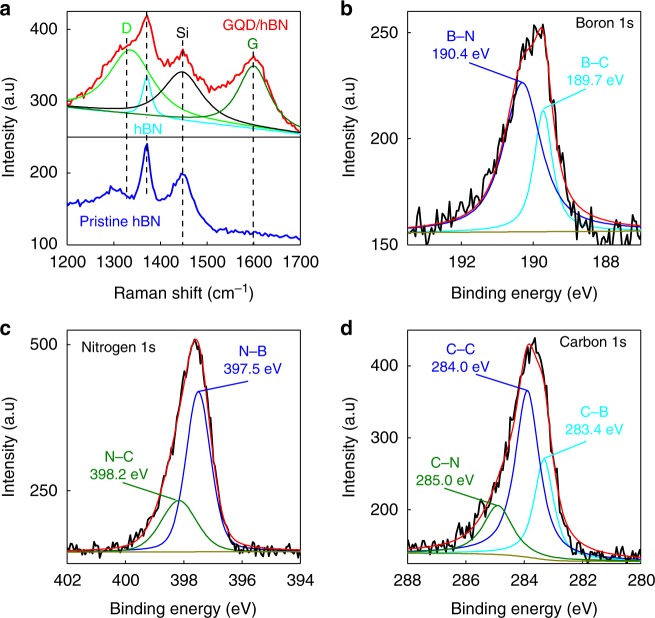
Characterisation of GQD-hBN interface of the in-plane heterostructure with GQDs of the size of 7 nm. **a** Raman spectra of GQD-hBN planar heterostructure (red) and pristine hBN (blue). XPS spectra of GQD-hBN planar heterostructure: **b** boron 1s, **c** nitrogen 1s and **d** carbon 1s spectrum

The formation of GQDs was confirmed by Raman spectroscopy (Fig. 3a) and electron energy loss spectroscopy (EELS) (Supplementary Figure 5). Typical Raman signals of GQDs and hBN were observed from the in-plane heterostructure of GQD-hBN transferred onto a SiO_2_ substrate: the D (1345 cm^−1^) and G (1595 cm^−1^) bands of graphene with an intervening E_2g_ peak (1371 cm^−1^) of an hBN^19,20^. Furthermore, the in-plane graphene domain size (*L*_*a*_) was calculated using the ratio of the integrated intensity (*I*_D_*/I*_G_) according to the Tuinstra–Koenig relation^21^ (Supplementary Table 1 and Supplementary Note 1), which is consistent with the size of GQDs, observed by SEM, Fig. 2. The formation of GQD-hBN heterostructures was also confirmed from absorption bands of hBN and GQDs in the UV-vis absorption spectra (Supplementary Figure 6) and EELS mapping (Supplementary Figure 5).

In order to characterise the interface between the GQDs and hBN in our in-plane heterostructures, we performed an XPS, Fig. 3b–d, which suggests a reasonable formation of bonds among carbon, nitrogen and boron atoms^22,23^. The obtained boron 1s peak, illustrated in Fig. 3b can be deconvoluted into two peaks with the energies of 189.7 eV and 190.4 eV, which are attributed to the B–C and B–N bonds, respectively. Notably, such a peak value of the B–N bond is very close to the measured value of boron 1s (190.5 eV) in the pristine hBN monolayers (see Supplementary Figure 1). Next, the nitrogen 1s peak, presented in Fig. 3c is composed of two peaks, corresponding to N–B bonds (397.5 eV) and N-C bonds (398.2 eV). Finally, the formation of C–B (283.2 eV) and C–N (285.0 eV) bonds were confirmed in the XPS carbon 1s spectrum (Fig. 3d). In addition, we also confirmed the C–N bonds at 1273 cm^−1^ and the B–N bonds at 1375 cm^−1^ in the measured infrared (IR) spectra of the GQD-hBN in-plane heterostructure (Supplementary Figure 7). The B–C bonds, which are expected to appear at ~ 1020 cm^−1^, were not identified owing to an emergence of a very strong SiO_2_ peak. Owing to the small size of our quantum dots—the lattice mismatch between graphene and hBN is not expected to lead to the formation of dislocations as in the case of bulk graphene/hBN planar heterostructures^24^.

### Vertical single-electron tunnelling transistors

**Fig. 4:**
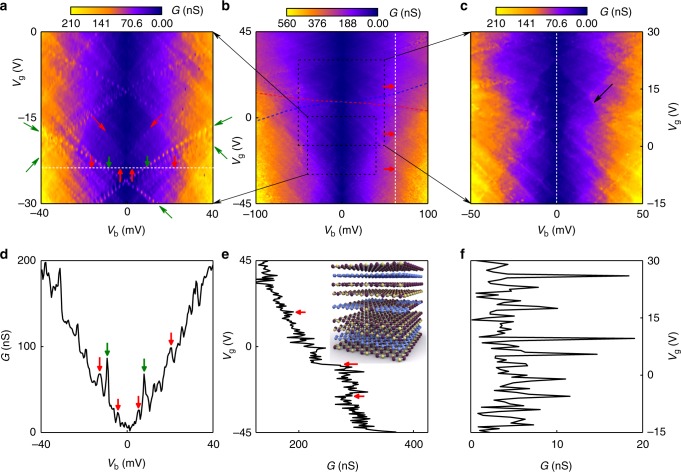
Multi-channel single-electron tunnelling transistors based on the GQDs of 13 nm in size. **a** The low excitation measurements of low bias region of **b**. Thin and long green arrows indicate the tunnelling events through the localised states in the middle hBN layer. Thick and short arrows indicate the edges of the Coulomb diamonds (red), and the resonances from localised impurity states (green) crossing the white dashed line (*G*(*V*_g_ = −24V, *V*_b_), presented on **d**). **b** Conductance *G*(*V*_g_,*V*_b_) measured at *T* = 250 mK. The red (blue) dashed line mark the event of the Fermi level in the top (bottom) graphene layer aligning with the Dirac point. Red arrows indicate the edges of the Coulomb diamonds crossing the white dashed line (*G*(*V*_g_, *V*_b_ = 63 mV), presented on **e**). **c** The magnified plot of **b** denoting the peculiar shape of Coulomb diamonds when the Fermi level in one of the graphene contacts aligns with the Dirac point (black arrow). The dashed white line indicates the cross-section presented in **f**. **d** The plot of conductance *G*(*V*_g_ = −24V,*V*_b_) from **a**. Thick and short arrows indicate the same events as in **a**: the edges of the Coulomb diamonds (red), and the resonances from localised impurity states (green). **e** The conductance *G*(*V*_g_, *V*_b_ = 63 mV) plot. Red arrows indicate the same events as in **b**: edges of the Coulomb diamonds. The inset shows the schematic structure of the van der Waals stack. Graphene contacts are separated by hexagonal boron nitride layers from the GQD-hBN layer. Carbon atoms are blue, boron—yellow, nitrogen—purple. **f** The conductance *G*(*V*_g_, *V*_b_ = 0 mV) plot from **c** (marked by white dashed line)

Such GQDs embedded in an hBN matrix are ideally suited for the formation of van der Waals heterostructures^25–27^. To this end, we prepared vertical tunnelling single-electron transistors^28–31^, where the transparency of the contacts is controlled by tunnelling through atomically thin hBN layers, thus avoiding the issue of the localised states in the contacts^8^. Our van der Waals heterostructures of the type 30 nm_hBN/Gr/2hBN/GQD-hBN/2hBN/Gr/20 nm_hBN have been assembled on Si/SiO_2_ substrate (acting as a back gate) by using dry transfer method^32^ (see device fabrication in Methods). Here, 30 nm_hBN stands for the hBN layer with an approximate thickness of 30 nm, Gr—for graphene, 2hBN—for 2-layer thick exfoliated hBN, GQD-hBN—for GQD-hBN lateral heterostructure. Schematic structure and the layer arrangement of our devices is presented on the inset to Fig. 4e. The GQD-hBN layer was sandwiched between two thin hBN layers to isolate the quantum dots from the contacts to ensure a long lifetime of electrons within the quantum dots, thus, to allow the detection of the single-electron energy levels. All our devices were fabricated in a symmetric configuration—with the same number of hBN layers on each side of the GQD-hBN layer. Devices with two (2hBN) and three (3hBN) layer thick hBN-tunnelling barriers have been produced. Information about all the devices measured can be found in the Supplementary Table 2.

We performed tunnelling spectroscopy on our van der Waals heterostructures, by applying a mixed signal of AC and DC bias voltages between the two graphene electrodes, and a gate voltage to the silicon substrate (see Methods and ref. ^33^ for details). Figure 4 presents a colour map of differential conductance *G*(*V*_g_, *V*_b_) = d*I*/d*V*_b_ as a function of the gate (*V*_g_) and bias (*V*_b_) voltages for one of our devices with 13 nm GQDs (examples of the tunnelling conductance for devices with 7 nm GQD are presented in Supplementary Figure 8). Two types of sharp peaks can be identified on top of the smooth background; (i) those organised into overlapping diamonds (the edges of the diamonds are marked by thin long red arrows, Fig. 4a), (ii) those following square root dependence (marked by thin long green arrows, Fig. 4a–c). We attribute the peaks with square root dependence in *G*(*V*_g_, *V*_b_) to arise owing to tunnelling through the localised electronic state, and the diamond-shaped features—to Coulomb blockade diamonds owing to tunnelling through single-electron states in individual GQDs.

Each diamond corresponds to a Coulomb blockade regime in one particular GQD. For a single GQD, one would observe a sequence of diamonds that connect to each other only at vertices. As we have a large number of GQDs connected in parallel—we observe a number of overlapping diamonds^34^. The zero-bias conductance within the diamond, Fig. 4f is given by the background tunnelling through the five-layer hBN (two layers of hBN on each side of the middle GQD-hBN layer) and is within the expected range^35^, indicating the absence of the pin-holes in the barrier. To confirm that Coulomb diamonds are originating from the single-electron charging events happening at GQDs, heterostructures of the stack 30 nm_hBN/Gr/2hBN/CVD hBN/2hBN/Gr/20 nm_hBN were produced. In such devices, the same CVD-grown hBN as used in devices of Fig. 4 is utilised, however, this CVD hBN does not contain any GQD in it. No Coulomb diamonds were observed in such devices (see Supplementary Figure 9). Furthermore, the conductivity of such devices is significantly lower than that for devices with GQDs, which proves that the Coulomb diamonds we observe are indeed coming from the GQDs.

In our diagrams (Fig. 4), each set of diamonds corresponds to a particular graphene quantum dot. Thus, we can estimate the number of GQDs involved in tunnelling, which gives us ~ 40 quantum dots connected in parallel for the device with 13 nm GQD. Thus, the number of GQDs we see participating in tunnelling is much smaller than the total number of GQDs within the area of the device (~ 50 GQDs per µm^2^, the total area of the device ~30 µm^2^ for the sample with 13 nm GQD). Currently we don’t have an explanation for this effect. However, it can be speculated that as it is the silicon gate (separated from the layer with GQD by ~ 300 nm of SiO_2_ and hBN), which provides the most efficient screening (graphene electrodes provide only weak screening)—GQD interact strongly between themselves via Coulomb interaction. It means that the observed Coulomb diamonds are the result of the collective behaviour of several GQDs within the 300 nm radius. This would also explain the different intensities of the conductivity peaks, Fig. 4d, f.

### Low-density GQDs

**Fig. 5:**
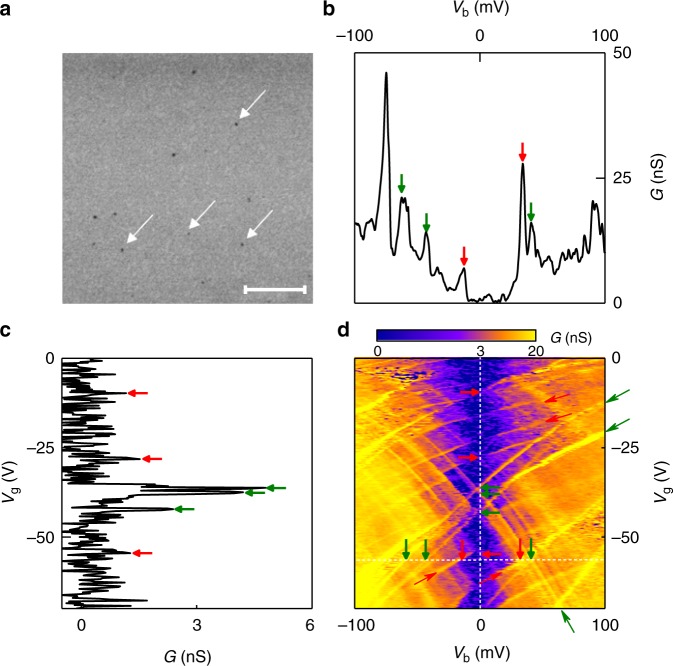
Low-density non-periodic array of 10 nm GQDs embedded in hBN matrix and electron tunnelling transistors based on such GQDs. **a** SEM image of a GQD-hBN sample obtained after the transfer of hBN monolayer on Pt NPs/SiO_2_ substrate and the conversion reaction. It shows GQDs with a long spacing (0.5–1.5 μm), marked by white arrows. Scale bar 1 μm. **b** The conductance *G*(*V*_g_ = −56V,*V*_b_) plot (extracted from **d** along the horizontal white dashed line). Arrows indicate peaks originating from boundaries of Coulomb diamonds (red) and from impurity-assisted tunnelling (green). **c** The conductance *G*(*V*_g_, *V*_b_ = 0 mV) plot (extracted from **d** along the vertical white dashed line). Arrows indicate peaks originating from boundaries of Coulomb diamonds (red) and from impurity-assisted tunnelling (green). **d** Conductance *G*(*V*_g_,*V*_b_) for a device with aperiodic 10 nm GQDs measured at *T* = 250 mK. Thick and short arrows indicate the crossing of the edges of the Coulomb diamonds (red arrows) and the impurity-assisted tunnelling peaks (green) with the white dashed lines. The positions of the arrows are the same as on **b** and **c**. Thin and long arrows indicate the edges of the Coulomb diamonds (red arrows) and the impurity-assisted tunnelling peaks (green arrows)

In order to avoid the large number of GQDs to be connected in parallel, thus obscuring the Coulomb diamonds, hBN layer with a low density of graphene islands was prepared. To this end, we used a strongly diluted micellar solution to achieve a low concentration of H_2_PtCl_6_: 1 mL of the PS-P4VP copolymer solution with H_2_PtCl_6_ was diluted by 400 mL of pure PS-P4VP. Such mixed solution was spin-coated on the SiO_2_ substrate, and the micellar film was treated by oxygen plasma to produce Pt NPs. The hBN monolayer was transferred onto the Pt NPs/SiO_2_ substrate, and the conversion reaction was performed for conversion of hBN on Pt NPs to graphene. After the aqua regia treatment to remove Pt NPs, a GQD-hBN monolayer with a relatively long spacing (0.5–1.5 μm) between GQDs (Fig. 5a) was obtained. Note, that this method gives a non-uniform distribution of GQDs.

We used such hBN with a low density of GQDs to prepare single-electron tunnelling transistors 30 nm_hBN/Gr/2hBN/GQD-hBN/2hBN/Gr/20 nm_hBN as it has been described above (see inset to Fig. 4e). The conductance of one of such devices (with the active area of 30 µm^2^) as a function of the gate and bias voltages is presented in Fig. 5b–d. Note, that the characteristic conductance for such a device is at least an order of magnitude smaller than that for devices with periodic, high-density arrays of GQDs (see the data presented on Fig. 4, note that the areas and the barrier thickness for these devices are the same). This is because the tunnelling now occurs through a smaller number of GQDs. At the same time, the Coulomb diamonds are visible much clearer in such devices (Fig. 5d) partly because of smaller number of overlapping Coulomb diamonds owing to smaller number of GQD, and partly because each GQD now act independently, interacting only weakly with other GQDs, as the distance between them is larger than the distance to the gate.

## Discussion

**Fig. 6:**
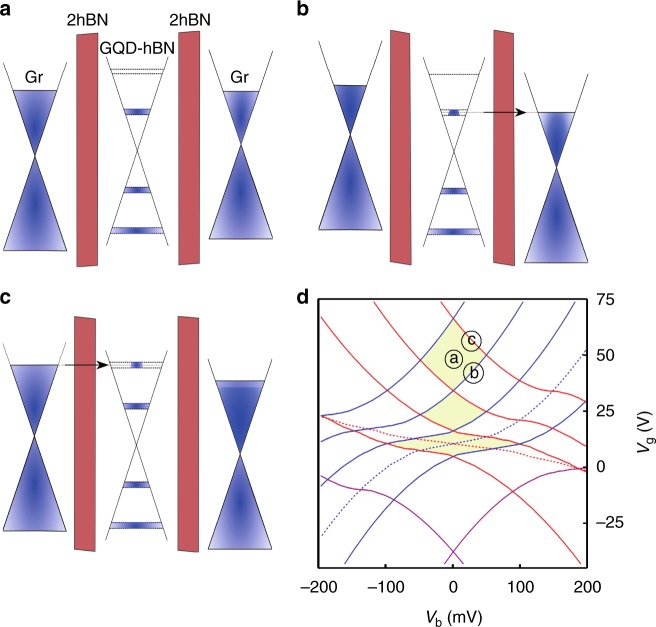
Modelling of the single-electron charging effect. **a**–**c** Schematic representation of a single-electron charging effect. The corresponding electrostatic lines are denoted in **d**. **d** Modelling example of the alignment of the different energy levels in a device with 13 nm GQD (see Methods and Supplementary Notes 2 for the details of the modelling). Red (blue) dashed lines—Fermi level in top (bottom) graphene electrode aligning with the Dirac point. Purple lines—Fermi levels in the graphene contacts being aligned with the localised state located in the middle hBN layer with energy 140 meV below the Dirac point. The set of solid red (blue) lines correspond to single-electron energy levels in GQD aligning with the Fermi level in the top (bottom) electrode. Space between four of such lines forms a Coulomb blockade diamond. Note the distorted shape of the diamond when the Fermi level in the contacts passes through the Dirac points

The schematics of the formation of the diamonds are presented in Fig. 6a–c and Supplementary Figures 11 and 12. When the size quantisation levels in the GQD are positioned outside of the bias window—no current flows through the quantum dots. At positive biases, a finite conductance is observed once the size quantisation level is below the Fermi level in the top graphene (such events are modelled by red lines in Fig. 6d) and above the Fermi level in the bottom graphene (modelled by blue lines in Fig. 6d). The combination of four of such lines gives a diamond of low conductivity. If the Fermi level in one of the graphene contacts is close to the Dirac point, where the density of states vanishes—the electrostatics dictates^30^ that the edges of the diamonds will not be straight lines anymore and will have square root dependence in the *V*_g_−*V*_b_ coordinates (see Fig. 6d and the Supplementary Note 2 for the details of the model). Such events are indeed observed in our measurements (marked by the black arrow in Fig. 4c, also see Supplementary Note 2 and Supplementary Figure 10).

The width of the diamonds in *V*_b_ gives a characteristic charging energy required to place an extra electron into a quantum dot. Experimentally, the easiest way to determine the width of the diamonds is by taking constant bias cross-sections (such as presented in Fig. 4e) of the conductivity plot *G*(*V*_g_, *V*_b_), where the edges of the diamonds are seen as distinct steps (marked by red arrows on Fig. 4b, e). The bias at which such steps disappear is then taken as the width of the diamonds. For our 13 nm (Fig. 4), 10 nm (Fig. 5) and 7 nm (Supplementary Figure 8a, b) quantum dots the charging energy was found to be of the order of 80 ± 15 meV, 100 ± 15 meV and 160 ± 20 meV, respectively, well in line with what is expected for the size quantisation for quantum dots of such a diameter^36–38^. The fact that the size quantisation energy scales as expected with the size of the GQD serves as an additional argument that the tunnelling occurs through the states in the quantum dots.

Simultaneously with the characteristic diamonds, a few conductance peaks that approximately follow the square root behaviour have been observed. The square root behaviour is coming from the linear density of states in the graphene electrodes and the fact that, owing to the small density of states the bottom graphene electrode does not entirely screen the electric field from the gate^39^. We attribute these features to the tunnelling through localised states in the central GQD layer^40,41^. Each localised state produces two lines in *G*(*V*_g_, *V*_b_)—when it aligns with the Fermi level in the bottom and in the top graphene contacts. The energy positions of these lines can be fitted with very high precision (Fig. 6d). From such a fitting we can extract the energy position of the localised states with respect to the Dirac points in the graphene layers as well as their spatial positions in the barrier. Thus, we found that energetically all the localised states observed are situated in the range of ± 150 meV in the vicinity of the Dirac points in the contacts. Our fitting also confirms that spatially all the localised states are indeed located in the central layer^42^ (hBN with GQDs). Note that it has been demonstrated that impurity states in the middle of the barrier contribute the strongest to the tunnelling current^43^ (see Supplementary Figure 13 for examples of phonon- and impurity-assisted tunnelling). The number of localised states we can see in our devices is very low (between 3 and 6, depending on the particular device, see Supplementary Table 2) much lower than the number of the GQD observed. This suggests that the edges of our GQDs are well passivated and do not produce additional localised states.

In conclusion, we demonstrated a way of synthesis of GQDs embedded in the hBN matrix. Such GQDs exhibit a very low number of edge states. The geometry allows easy incorporation into van der Waals heterostructures, where we demonstrate single-electron tunnelling transistors. Our approach—the combination between the in-plane and van der Waals heterostructures—allows the fabrication of high-quality GQDs for transport experiments. The in-plane heterostructures allow fabrication of GQDs without the dangling bonds and localised states at the perimeter. At the same time, the van der Waals heterostructures allow fabrication of controlled tunnelling barriers, again without any localised states. We hope that our approach will pave the way for many other types of devices and physical phenomena to be studied.

## Methods

### The growth of GQDs embedded in the hBN sheet

The single layer of hBN was synthesised on Pt foil using ammonia borane as a precursor by the CVD method. Experimental details on the synthesis of CVD-grown hBN on Pt can be found in a previous report^17^. The Pt NPs array on a SiO_2_ substrate was prepared using self-patterning diblock copolymer micelles^18^. A single layer of PS-P4VP micelles with H_2_PtCl_6_, a precursor for Pt NPs, in their cores was spin-coated on the SiO_2_ substrate. To fabricate the Pt NP array, the micellar film on the SiO_2_ was annealed at 400 °C for 30 min in air. The hBN layer was transferred onto the Pt NPs/SiO_2_ substrate using a wet-transfer method (electrochemical delamination). Then, the hBN layer transferred on Pt NPs/SiO_2_ was loaded into the centre of a vacuum quartz tube in a furnace for the conversion reaction^16^. The tube was pumped down to 0.21 Torr with pure argon gas (50 sccm). Then the furnace was heated to 950 °C in 40 min. During the reaction, methane gas (5 sccm) with argon (50 sccm) was flown as the source for graphene growth. During the reaction, the hBN region on the Pt NPs was converted to graphene, and after 10 min of growth, a uniform GQD array embedded in the hBN film was obtained.

### Transfer method

The GQD-hBN film on Pt NPs/SiO_2_ could be transferred to any other substrate via wet-transfer method using HF and an aqua regia solution. First, polystyrene (PS) was spin-coated on the sample, and it was immersed in an hydrogen fluoride solution (5% in deionised water) to detach the GQD-hBN film. The floating PS film was then transferred to the aqua regia solution (3:1 mixture of hydrochloric acid and nitric acid) to remove Pt NPs and then rinsed with copious amounts of DI water. Finally, the film was transferred onto the target substrate and PS was removed with toluene to obtain a GQD-hBN film on the substrate.

### Characterisation

SEM (Verios 460, FEI) and atomic force microscopy (Dimension Icon, Bruker) were used to determine the surface morphology of the samples. Raman spectra were measured using a micro Raman spectroscope (alpha 300, WITec GmbH) using 532 nm. The UV-vis absorption spectra of the GQD-hBN samples were recorded on a Cary 500 UV-vis-near IR spectroscope, Agilent. XPS (K-Alpha, Thermo Fisher) and Nano-FTIR (neaSNOM, aspect) were performed to determine the composition of the GQD and confirm the formation of an interface between GQD and hBN. Low-voltage Cs aberration-corrected transmission electron microscopy (Titan Cube G2 60-300, FEI), operated at 80 kV with a monochromated electron beam, was used for EELS analysis. The spatial and energy resolutions for the EELS measurement are 2 nm and 1.5 eV, respectively.

### Device fabrication

Optical images of the flakes at different stages of the fabrication process are presented on Supplementary Figure 14. The electrical response of the GQDs embedded in monolayer hBN was investigated by assembling vertical tunnel van der Waals heterostructures consisting of the stack of 30 nm_hBN/Gr/2hBN/GQD-hBN/2hBN/Gr/20 nm_hBN placed on an oxidised silicon wafer (300 nm of SiO_2_). Here, the bottom and top layers of hBN were used for the purpose of encapsulation. As the GQDs are embedded in the large area monolayer hBN on Si/SiO_2_, the vertical heterostructure was assembled in two halves by adopting a mix of dry and wet flake transfer procedure: first, a stack of Si/ SiO_2_/30 nm_hBN/Gr/2hBN was prepared by standard flake exfoliation and dry transfer procedure. To prepare the other half, a stack of 2hBN/Gr/20 nm_hBN was prepared by the dry pick up procedure using a polymethyl methacrylate (PMMA) membrane. This stack on the membrane was aligned and dropped on the GQD-hBN/SiO_2_/Si substrate. To release the stack from Si/SiO_2_, 8% PMMA was spun on the sample and Si/SiO_2_ was etched using potassium hydroxide (KOH) solution. The floated membrane was thoroughly rinsed with DI water several times to remove KOH residues from the membrane. Finally, to complete the device, this membrane containing GQD-hBN/2hBN/Gr/20 nm_hBN was dropped on the stack prepared in the first half.

For electrical characterisation of this vertical heterostructure, Cr/Au edge contacts were made on the top and bottom graphene layers using electron beam lithography followed by boron nitride etching, metal deposition and lift-off process. Electrical characterisation of graphene contacts is presented in Supplementary Figure 15. Additional tunnelling measurements with Coulomb diamonds clearly marked are presented in Supplementary Figure 16.

## Supplementary information

Supplementary Information

## Data Availability

The data that support the findings of this study are available from the corresponding author upon reasonable request.
