# Author Correction: Planar and van der Waals heterostructures for vertical tunnelling single electron transistors

**DOI:** 10.1038/s41467-019-08910-x

**Published:** 2019-02-25

**Authors:** Gwangwoo Kim, Sung-Soo Kim, Jonghyuk Jeon, Seong In Yoon, Seokmo Hong, Young Jin Cho, Abhishek Misra, Servet Ozdemir, Jun Yin, Davit Ghazaryan, Matthew Holwill, Artem Mishchenko, Daria V. Andreeva, Yong-Jin Kim, Hu Young Jeong, A-Rang Jang, Hyun-Jong Chung, Andre K. Geim, Kostya S. Novoselov, Byeong-Hyeok Sohn, Hyeon Suk Shin

**Affiliations:** 1 Department of Energy Engineering, Ulsan National Institute of Science & Technology (UNIST), Ulsan, 44919 Republic of Korea; 2 Department of Chemistry, Seoul National University, Seoul, 08826 Republic of Korea; 3 Department of Chemistry, UNIST, Ulsan, 44919 Republic of Korea; 4 Department of Physics, Konkuk University, Seoul, 05029 Republic of Korea; 5 School of Physics and Astronomy, University of Manchester, Manchester, M13 9PL United Kingdom; 6 Department of Physics, Indian Institute of Technology Madras, Chennai, 600036 India; 7 Department of Physics, National Research University Higher School of Economics, Staraya Basmannaya 21/4, Moscow, 105066 Russian Federation; 8 Department of Materials Science and Engineering, National University of Singapore, Singapore, 117575 Singapore; 9 Center for Multidimensional Carbon Materials, Institute of Basic Science (IBS), Ulsan, 44919 Republic of Korea; 10 UNIST Central Research Facilities (UCRF), UNIST, Ulsan, 44919 Republic of Korea; 11 Low Dimensional Carbon Material Center, UNIST, Ulsan, 44919 Republic of Korea; 12 Present Address: Carbon Composite Materials Research Center, Korea Institute of Science and Technology (KIST), Wanju, 55324 Republic of Korea

Correction to *Nature Communications*; 10.1038/s41467-018-08227-1, published online 16 January 2019

The original version of this Article contained an error in the spelling of the author Matthew Holwill, which was incorrectly given as Mathew Holwill. This has now been corrected in both the PDF and HTML versions of the Article.

