# Supplementary material for: Planar and van der Waals heterostructures for vertical tunnelling single electron transistors
Source: Nat Commun. 2019 Jan 16;10:230. doi: 10.1038/s41467-018-08227-1 (PMC6335417; doi:10.1038/s41467-018-08227-1)
Supplement: Supplementary file 1 — Supplementary Information [file 41467_2018_8227_MOESM1_ESM.pdf]

Supplementary Information

Planar and van der Waals heterostructures for vertical tunnelling single electron transistors

Gwangwoo Kim[1], Sung-Soo Kim[2], Jonghyuk Jeon[2], Seong In Yoon[1], Seokmo Hong[3], Young Jin Cho[4], Abhishek Misra[5,6], Servet Ozdemir[5], Jun Yin[5], Davit Ghazaryan[5,7], Mathew Holwill[5], Artem Mishchenko[5], Daria V. Andreeva[8], Yong-Jin Kim[9], Hu Young Jeong[10], A-Rang Jang[1,3], Hyun-Jong Chung[4], Andre K. Geim[5], Kostya S. Novoselov[5], Byeong-Hyeok Sohn[2] and Hyeon Suk Shin[1,3,9, 11]

[1]Department of Energy Engineering, Ulsan National Institute of Science & Technology (UNIST), Ulsan 44919, Republic of Korea

[2]Department of Chemistry, Seoul National University, Seoul 08826, Republic of Korea

[3]Department of Chemistry, UNIST, Ulsan 44919, Republic of Korea

[4]Department of Physics, Konkuk University, Seoul 05029, Republic of Korea

[5]School of Physics and Astronomy, University of Manchester, Manchester M13 9PL, United Kingdom

[6]Department of Physics, Indian Institute of Technology Madras, Chennai, India

[7]Department of Physics, National Research University Higher School of Economics, Staraya Basmannaya 21/4, Moscow 105066, Russian Federation

[8]Department of Materials Science and Engineering, National University of Singapore, Singapore, 117575, Singapore

[9]Center for Multidimensional Carbon Materials, Institute of Basic Science (IBS), Ulsan 44919, Republic of Korea

[10]UNIST Central Research Facilities (UCRF), UNIST, Ulsan 44919, Republic of Korea

[11]Low Dimensional Carbon Material Center, UNIST, Ulsan 44919, Republic of Korea

Correspondence and requests for materials should be addressed to H. S. S. (email: shin@unist.ac.kr), B. H. S. (email: bhsohn@snu.ac.kr) and K. S. N. (email: kostya@manchester.ac.uk).

Supplementary Figures

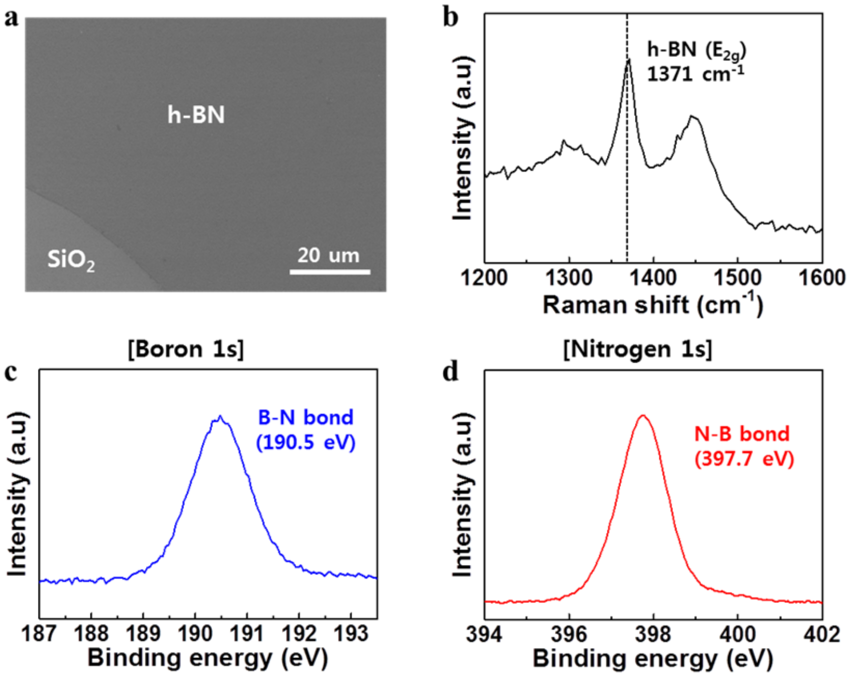

Supplementary Figure 1. a, b, The SEM image and Raman spectrum of the CVD grown pristine hBN monolayer. **c, d,** The corresponding XPS spectra: (c) Boron 1s, and (d) Nitrogen 1s.

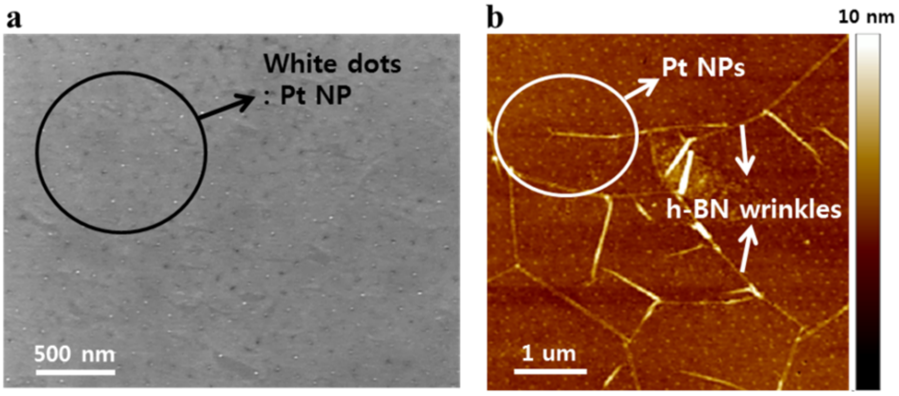

Supplementary Figure 2. a, b, The SEM and AFM images of the as-grown layer of GQD-hBN on an array of Pt NPs (7nm) spread over SiO_2 substrate.

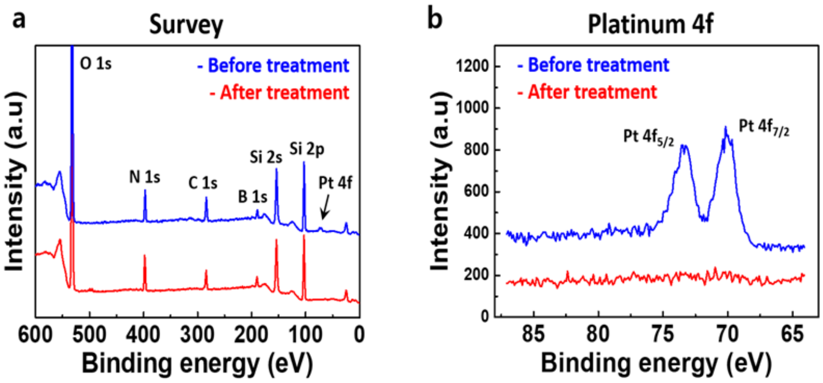

Supplementary Figure 3. The XPS spectra of GQD-hBN planar heterostructure on SiO_2 substrate. a, Survey, and **b,** Pt 4f spectra. Blue and red spectra are for as-prepared GQD/hBN on Pt NPs/SiO_2 substrate (before the aqua regia treatment) and the GQD-hBN after the aqua regia treatment to remove Pt NPs, respectively.

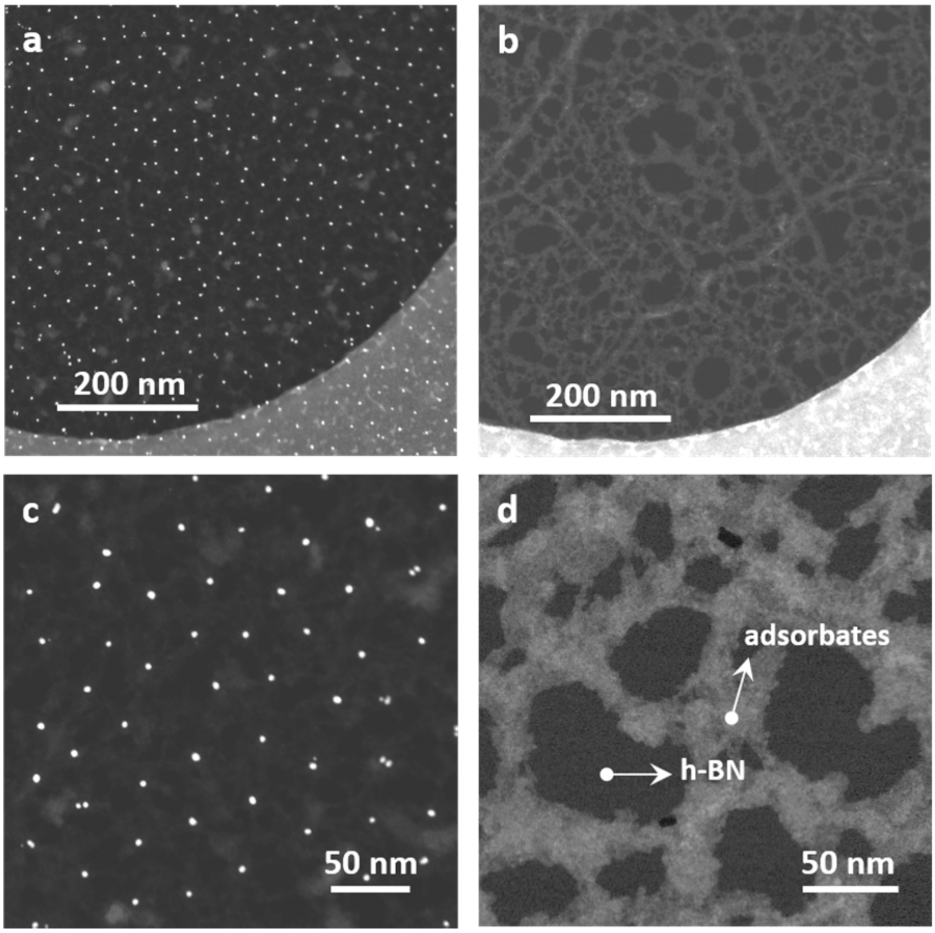

Supplementary Figure 4. TEM images of GQD-hBN. a,c Pt NPs in GQD-hBN. **b,d** The GQD-hBN after the aqua regia treatment. There are no Pt NPs after the treatment. The white dots in (a,c) are Pt NPs.

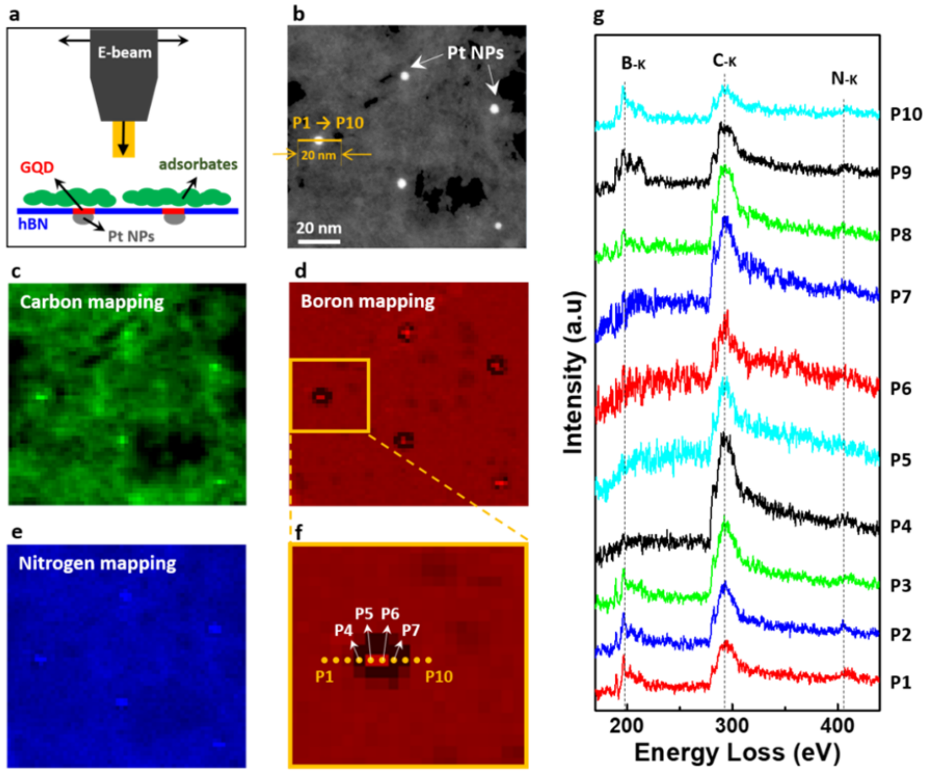

Supplementary Figure 5. EELS spectrum of GQD-hBN. a Schematics of EELS mapping of GQD-hBN on Pt NPs/SiO_2. **b**, TEM image of GQD-hBN on Pt NPs. The white dots are 7 nm Pt NPs. **c-e**, Corresponding EELS mapping images of (**c**) carbon, (**d**) boron, and (**e**) nitrogen, respectively. **f**, A magnified image marked in **d**. The boron signal was not detected at P4 to P7 where the Pt NP exist. Note that the points P5 and P6 with the strong signal are due to the strong background of Pt signal because we could not completely subtract the strong Pt background. Note that boron signal is absent in P5 and P6 (see panel **g**). In the nitrogen mapping image, the N signal is too low to be detected (see *Nano Lett.* 2013, *13*, 1834). **g**, The EELS spectra were obtained at different positions (yellow line, P1 to P10) with 2 nm spatial resolution in **f** by subtracting the background of the Pt signal from the original EELS spectra. The peak for Boron is not detected in P4, P5, P6, and P7, indicating conversion of BN to graphene. Note that GQDs in **c** and **g** are not distinguishable from carbon signal of many adsorbates.

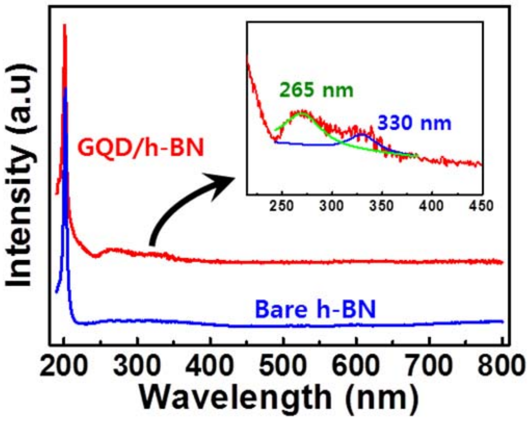

Supplementary Figure 6. The UV-vis absorbance spectra of pristine hBN and GQD-hBN planar heterostructure on quartz substrates. It shows the typical absorption band of hBN[1] located at 200 nm, and those of 265 nm and 330 nm for GQDs[2].

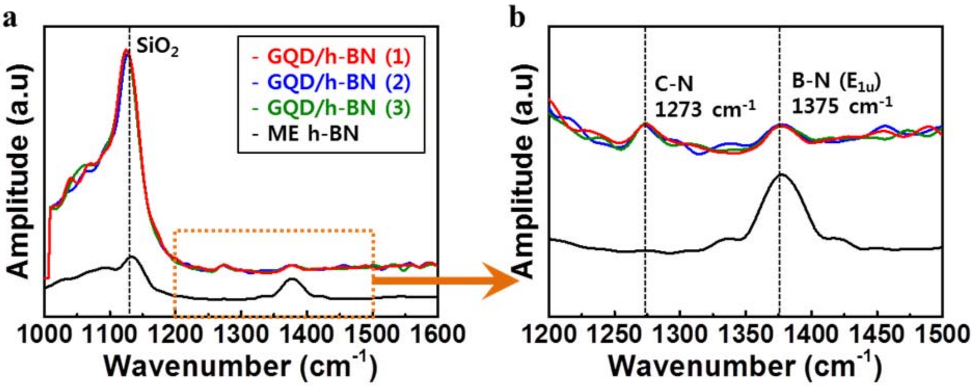

Supplementary Figure 7. a, The IR spectra for the GQD-hBN and mechanically exfoliated (ME) hBN (2nm thickness) on SiO_2 by means of AFM-IR. Three spectra at different points were measured for a GQD-hBN sample (1-3). **b,** Magnified spectra in the range of 1200-1500 cm^{-1}.

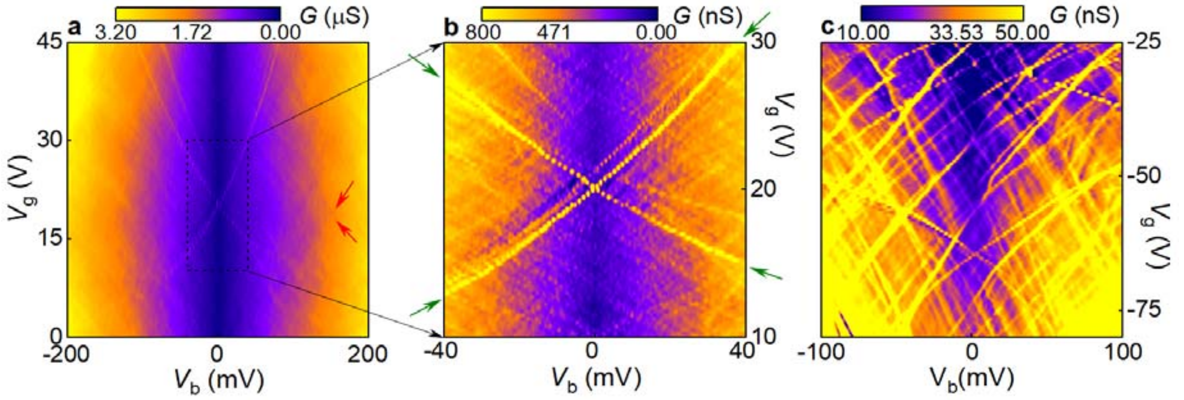

Supplementary Figure 8. The hBN/Gr/2hBN/GQD-hBN/2hBN/Gr/hBN multi-channel single electron tunnelling transistors based on GQDs embedded in the hBN matrix. a, Conductance $G(V_g, V_b)$ for the device with the GQDs of 7nm size prepared by the technique of self-assembly (measured at T=250mK). Red arrows indicate the edges of the Coulomb diamonds at the bias voltage of ≈160mV. **b,** The low excitation measurements of low bias region of (a), indicating the tunnelling events through the localised density of states in the middle hBN layer. The olive arrows denote the localised states with the energy 140meV below the Dirac point. **c,** Low-density non-periodic array of GQDs of 10nm size embedded in hBN layer placed in-between of hBN/Gr/2hBN/ and /2hBN/Gr/hBN heterostructures. T=250mK conductance $G(V_g, V_b)$ for a device with such GQDs and low quality exfoliated encapsulating bilayers of hBN. Because of the technology we used, there are roughly 4 times more 7nm (Supplementary Figure 10 (a)) GQD per unit area than there are 13nm (Figure 4 (b)) GQD, thus, there are more overlapping diamonds for 7nm GQD.

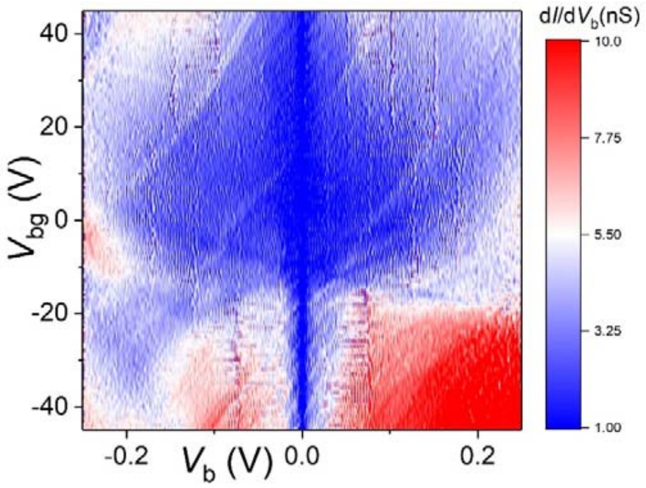

Supplementary Figure 9. T=250mK tunnelling conductance $G(V_b,V_g)$ of Si/SiO_2 substrate supported hBN/Gr/2hBN/CVD-hBN/2hBN/Gr/hBN heterostructure. The area of the device is 82μm^2. Note, the middle hBN monolayer was grown by CVD, but no GQD was formed on it. Note, significantly lower conductivity (even though the area of the device is significantly larger than for those presented in the main text) due to the absence of the additional conductance channels due to GQD. There is a small number of the impurity states, however, which might be originating from either defect in the CVD hBN, or due to contamination between the layers introduced during the fabrication.

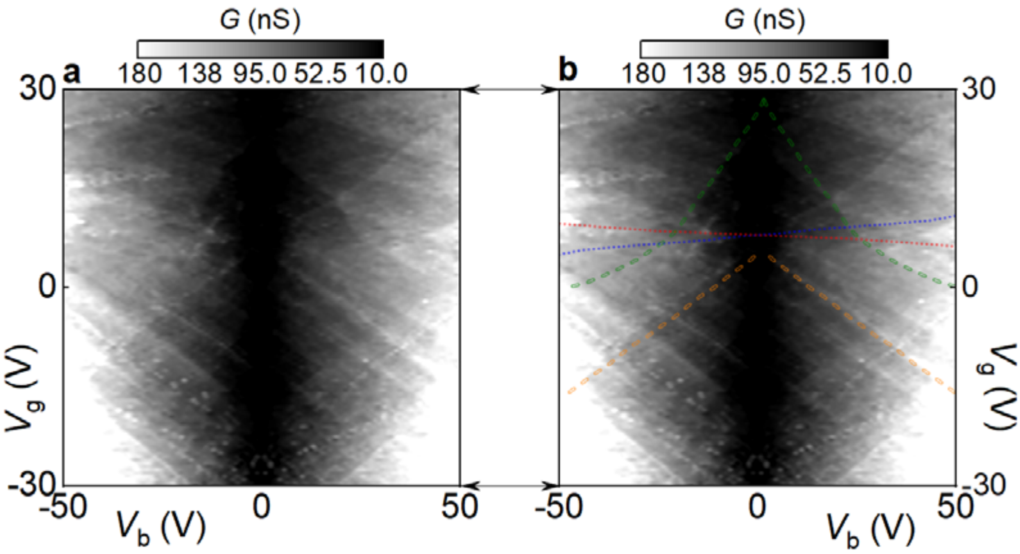

Supplementary Figure 10. **The peculiar shape of the Coulomb diamonds at low bias and gate voltages.** **a**, T=250mK low excitation G (V_b, V_g) for the device with 7nm GQDs embedded in the central layer of CVD hBN. **b,** same as (a), but denoting a peculiar constitution of the lines forming Coulomb diamonds when the Fermi levels in either graphene contacts align with the Dirac points (green dashed lines).On the contrary, Orange dashed lines indicate the usual shape of the constituent lines of Coulomb diamonds when the Fermi levels are away from the DPs of both graphene layers, blue and red dotted lines.

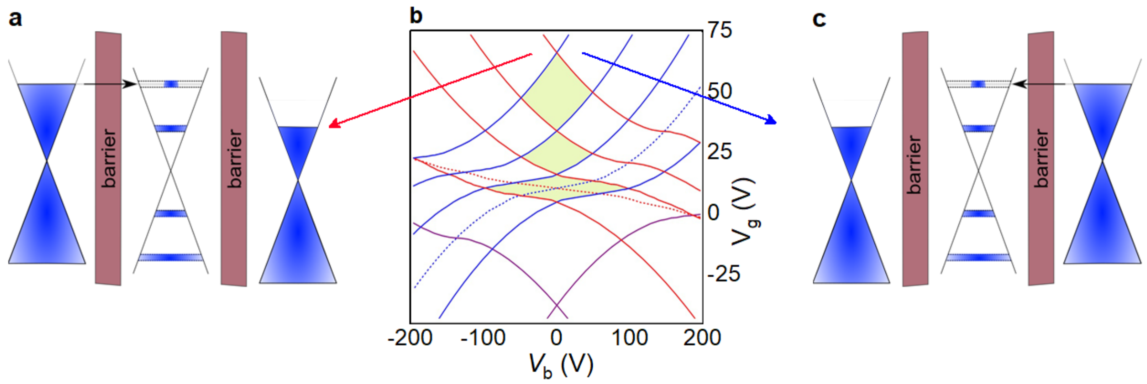

Supplementary Figure 11. Single electron charging effect model. a-c, Schematic representation of a single electron charging effect denoting particular approximations used in the modelling of electrostatic parameters of the resulting final heterostructure. **b,** same as Figure 6d of the main text.

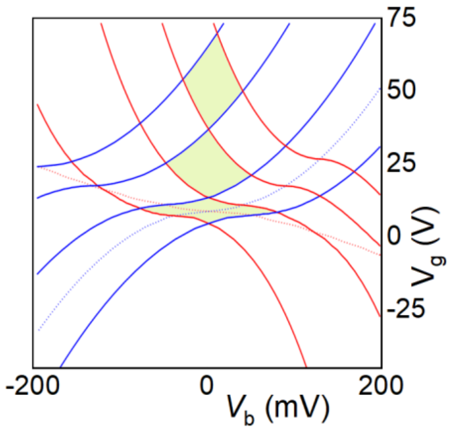

Supplementary Figure 12. Single electron charging effect model. Same as Figure 12b, except the chemical potential of the GQD-hBN layer is aligned with that in the source (bottom) electrode.

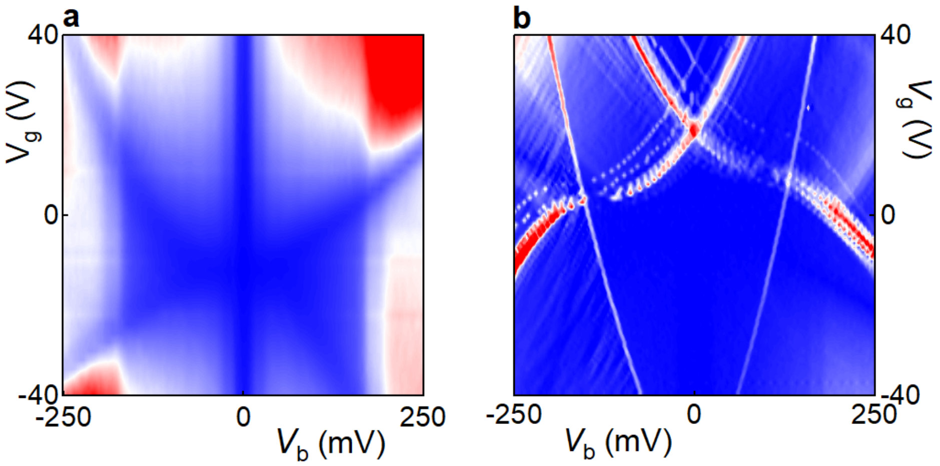

Supplementary Figure 13. ***T*=1.5K tunnelling conductance *G*(V_b, V_g) of Si/SiO_2 substrate supported Gr/hBN/Gr heterostructures. a,** Tunnelling through pristine hBN trilayer mounted in-between two graphene monolayers (colour scale is blue to white to red, 20nS to 2µS to 4µS). Dark Blue *X* shaped region corresponds to the event of the passage of chemical potential through DPs of graphene layers; vertical features represent phonon-assisted resonant tunnelling process[3]. **b,** Tunnelling through impurity states of low-quality tetralayer hBN mounted in-between monolayer graphene electrodes (colour scale is blue to white to red, 0nS to 20nS to 40nS). Three various peaks in conductance (red and white) correspond to the tunnelling through three different localised states and all follow the square root dependence[4]. Tunnelling conductance of the heterostructures of hBN/Gr/hBN/Gr/hBN without the additional layer of the CVD hexagonal boron nitride with embedded GQDs is shown in the Supplementary Figure 11. Here, in the case of pristine high-quality hBN spacer mounted in between graphene layers, resonant tunnelling features (straight vertical lines at fixed voltages of V_b) occur through the assistance of both acoustic and optical phonons[3]. Notably, apart from the X shaped low conductivity region, which corresponds to the event of the passage of the chemical potential through the Dirac points of graphene contacts, there are no other features observed. On the other hand, in Supplementary Figure 10 (b), it was demonstrated that if a low-quality hBN is used as a tunnelling barrier in similar heterostructure - the tunnelling is dominated by the impurity states that are located in the middle of the barrier.

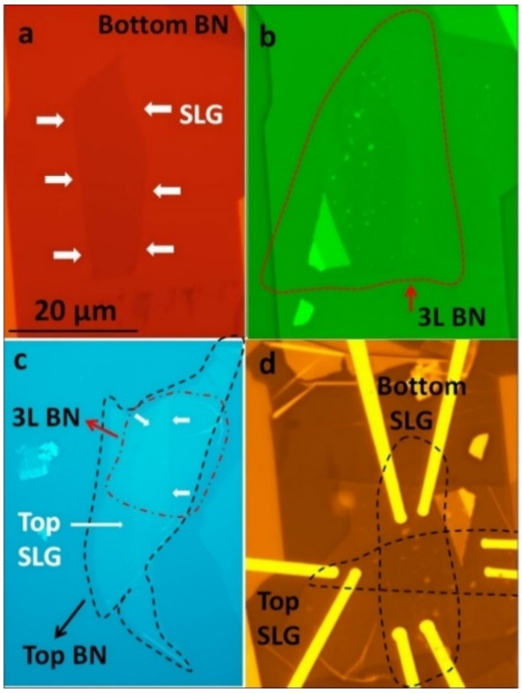

Supplementary Figure 14. Fabrication procedure for van der Waals tunnel heterostructure comprising the stack of 20nm_hBN/Gr/3L_hBN/GQD-hBN/3L_hBN/Gr/10nm_hBN. a, Single-layer graphene, indicated by white arrows, was transferred by flake peeling method on bottom hBN supported on Si/SiO_2 substrate. **b,** Trilayer hBN, outlined by red dashed line, was then transferred on graphene layer shown in (a). **c,** Separately a PMMA membrane was prepared with 10nm hBN, another graphene layer and trilayer hBN were picked up using this hBN. This PMMA membrane containing the stack of 3hBN/Gr/10_hBN was further aligned and dropped on GQD-hBN on Si/SiO_2. To release this stack from Si/SiO_2, wet transfer procedure following a standard KOH etching procedure was performed. **d,** Finally, the heterostructure of GQD-hBN/3hBN/Gr/10nm_hBN was aligned and transferred on 20nm_hBN /Gr/3hBN shown in (b). Contacts to the top and bottom graphene layers were made by standard electron beam lithography, as shown in (d). Scale bar shown in (a) is the same for all images.

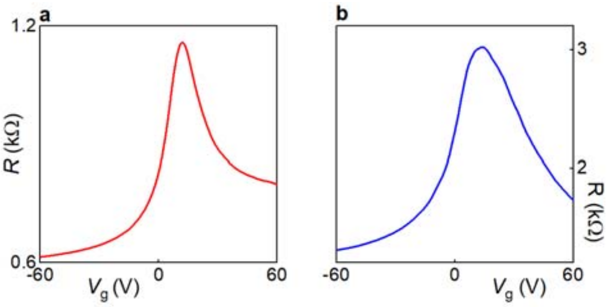

Supplementary Figure 15. **Resistance dependence of graphene on gate voltage for our van der Waals heterostructure with 13nm GQDs embedded in a central layer of hBN. a,** The 2-probe resistance measurements of the gate dependence of top monolayer graphene at *T*=0.25K. **b,** The 2-probe resistance measurements of the gate dependence of bottom monolayer graphene at *T*=0.25K.

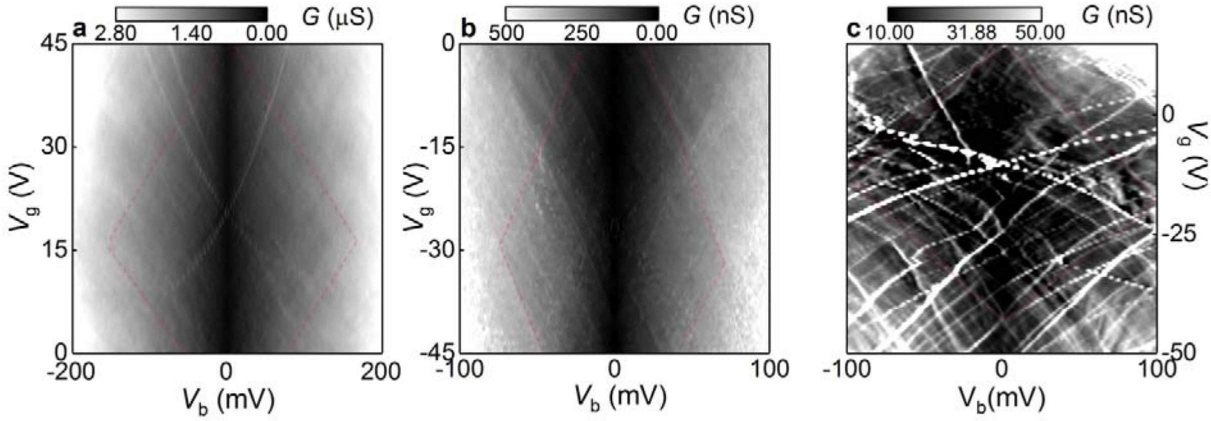

Supplementary Figure 16. **The hBN/Gr/2hBN/GQD-hBN/2hBN/Gr/hBN multi-channel single electron tunnelling transistors based on GQDs embedded in the hBN matrix with a guide to an eye emphasis of the Coulomb diamonds. a, C**onductance $G(V_g, V_b)$ for the device with the 7nm GQDs prepared by the technique of self-assembly (measured at T=250mK). The dashed red lines are guides to an eye, emphasising one of the Coulomb diamonds throughout the Figure. **b, S**ame as (a), except the device is with 13nm GQDs. **c, S**ame as (a), except the device is with low density non-periodic array of 10nm GQDs.

Supplementary Tables

Size of GQD (nm)	L_a (nm)
7 nm	8.98 nm
10 nm	10.12 nm
13 nm	11.79 nm

Supplementary Table 1. Graphitic domain size. The integrated intensity ratio, I_D/I_G, was used to determine the in-plane crystallite size L_a (nm) using the Tuinstra-Koenig relationship[5], as described in Supplementary Note 1 and Supplementary Equation (1).

Device N	Fabrication technique of hBN-GQD layer	Cross-sectional area	Number of hBN layers used as spacers	GQD size extracted from SEM	Number of prominent localised electronic states, extracted from tunnelling spectroscopy	Estimated number of GQDs involved in the tunnelling measurements	The estimated charging energy for the GQDs (approximation)
1	Self-assembly	60 ⍰m^2	3hBN	13nm	10	>80	80meV
2	Self-assembly	30 ⍰m^2	2hBN	13nm	3-4	~40	80meV
3	Self-assembly	32 ⍰m^2	2hBN	10nm	10	~40	100meV
4	Self-assembly	40 ⍰m^2	3hBN	7nm	5-6	~50	160meV
5	Pristine hBN	71 ⍰m^2	2hBN	N/A	1-2	N/A	0meV
6	Pristine hBN	80 ⍰m^2	2hBN	N/A	N/A	N/A	0meV
7	Non-periodic array	6 ⍰m^2	2hBN	10nm	10	~10	100meV
8	Non-periodic array	33 ⍰m^2	2hBN	10nm	6	~8	100meV

Supplementary Table 2. The functionality of the investigated devices.

Supplementary notes

Supplementary Note 1. Estimation of the size of the GQD from Raman measurements

We used the Tuinstra-Koenig relationship[5] to estimate the size of the GQD (L_a) from our Raman measurements:

$$L_a(\text{nm})=2.4\times10^{-10}\,\lambda^4\,(I_G/I_D) \tag{1}$$

Here λ is the wavelength of Raman excitation (532 nm), I_G and I_D – are the integrated intensities of the G and D peaks respectively.

Supplementary Note 2. Modelling the electrostatic parameters of final heterostructures

The electrostatic equations for the vertical Gr/hBN/GQD-hBN/hBN/Gr heterostructures could be evaluated similar to[4], but with specific modifications. Here, Gr stands for the bottom and top electrodes of monolayer graphene, hBN stands for mechanically exfoliated layers of hexagonal boron nitride, and GQD-hBN stands for the CVD layer of hBN with an embedded GQDs.

Note, that the difference in modelling tunnelling though localised states and through GQD is that we do not allow charge accumulation on the localised state and we allow such charge accumulation on GQD due to finite capacitance.

At first, for the modelling of the tunnelling events through the localised edge states in the middle GQD layer we consider a model of a three-plate capacitor with the n-doped Si and two monolayers of graphene. Accounting the fact that the electric field generated by the n-Si gate electrode is partially screened by the bottom graphene layer, owing to the linear spectrum, we obtain

$$\begin{cases} eV_b = \mu_B - \mu_T - e^2 d_{SP} n_T(\mu_T)/\varepsilon_0\varepsilon_{SP} \\ eV_g = \mu_B + e^2 d_{BG}(n_T(\mu_T) + n_B(\mu_B))/\varepsilon_0\varepsilon_{BG} \end{cases} \tag{2}$$

where d_{SP}, d_{BG}, ε_{SP}, ε_{BG} are the thicknesses and dielectric constants of the spacer and back gate insulating films, respectively, and ε_0 is the vacuum permittivity. The relation of carrier density and chemical potential is given as $n_i(\mu_i) = {\mu_i}^2/\pi\hbar^2 v_F^2$, where v_F is the Fermi velocity, and $\hbar$ is the Plank's constant. Considering the localised state as a level located at certain energy and a spatial position in the barrier developed by the gap of the spacer insulating film, we model resonant conditions of the tunnelling events through such states as

$$\mu_B = E_{LS} - (e^2 d_{SP} n_T(\mu_T)/\varepsilon_0\varepsilon_{SP})(d_{LS}/d_{SP}), \tag{3}$$

$$\mu_T = E_{LS} + (e^2 d_{SP} n_T(\mu_T)/\varepsilon_0\varepsilon_{SP})(d_{SP} - d_{LS}/d_{SP}), \tag{4}$$

where E_{LS} is the energy of the specific level, localised state, (counted from the zero state of unpthe erturbed system - neutrality point of bottom layer electrode), and d_{LS} is the spatial position of such a state in the barrier (counted from the bottom layer electrode).

Next, for the modelling of tunnelling events corresponding to the emergence of Coulomb diamonds due to the GQDs we consider the four-plate capacitor with n-Si, two monolayers of graphene, and middle GQD-hBN layer. Here, the GQD-hBN layer is introduced as an electrode with discrete energy levels (see Supplementary Figure 12), corresponding to the size quantization of GQDs. As indicated in the Supplementary Figure 12, there are two sets of lines; blue and red, corresponding to the different directions the single electron charging effect. To a good quantitative approximation, the chemical potential of the middle layer is considered to be aligned to the chemical potential of the top (drain) monolayer graphene electrode for both directions of the tunnelling (see Supplementary Figure 8a and red line in b). Such approximation allows for the analytical solution of the model. In such a scenario, accounting an additional screening arising from the middle GQD-hBN layer we obtain

$$\begin{cases} eV_b = \mu_B - \mu_M - e^2 d_{SP}(n_M(\mu_M) + n_T(\mu_T))/\varepsilon_0\varepsilon_{SP} \\ eV_g = \mu_B + e^2 d_{BG}\left(n_M(\mu_M) + n_B(\mu_B)\right)/\varepsilon_0\varepsilon_{BG} \\ \mu_M = \mu_T + e^2 d_{SP2}(n_T(\mu_T))/\varepsilon_0 \end{cases} \quad (5)$$

where $d_{SP2} = d_{SP}/2$, and μ_M is the chemical potential of the middle GQD-hBN layer. In this case, the resonant tunnelling condition for the tunnelling direction bottom to top are given as

$$\mu_B = (e^2 d_{SP2}(n_M(\mu_M) + n_T(\mu_T)/\varepsilon_0\varepsilon_{SP}) + E_i, \quad (6)$$

where E_i are the discrete energy levels in the middle GQD-hBN layer, and d_{SP2} is the thickness of the hBN barrier between the bottom graphene and GQD-hBN layer. Likewise, to model the resonant conditions for an opposite direction of the tunnelling, one needs to consider the relation between the chemical potential of the top layer of graphene and the emerged electrostatic field to the energy levels E_i of a middle GQD-hBN layer (see Supplementary Figure 12c, and blue lines in b).

To prove that our assumption of aligning the chemical potential of the GQD-hBN layer with that in the drain electrode does not introduce any significant qualitative errors, we modelled the opposite extreme situation, when the chemical potential of the GQD-hBN layer is aligned to that in the source (bottom) electrode. One can see from Supplementary Figure 13 that the position and the shape of the diamonds are very much the same as in the previous case. Thus, our approximate model, which could be solved analytically, provides a good qualitative and quantitative description of tunnelling through GQDs.

Supplementary References

1 Kim, G., Jang, A. R., Jeong, H. Y., Lee, Z., Kang, D. J. & Shin, H. S. Growth of High-Crystalline, Single-Layer Hexagonal Boron Nitride on Recyclable Platinum Foil. *Nano Letters* **13**, 1834-1839, doi:10.1021/nl400559s (2013).

2 Pan, D., Zhang, J., Li, Z. & Wu, M. Hydrothermal Route for Cutting Graphene Sheets into Blue-Luminescent Graphene Quantum Dots. *Advanced Materials* **22**, 734-738,

doi:doi:10.1002/adma.200902825 (2010).
3 Vdovin, E. E., Mishchenko, A., Greenaway, M. T., Zhu, M. J., Ghazaryan, D., Misra, A., Cao, Y., Morozov, S. V., Makarovsky, O., Fromhold, T. M., Patane, A., Slotman, G. J., Katsnelson, M. I., Geim, A. K., Novoselov, K. S. & Eaves, L. Phonon-assisted resonant tunneling of electrons in graphene-boron nitride transistors. *Phys. Rev. Lett.* **116**, 186603, doi:10.1103/PhysRevLett.116.186603 (2016).
4 Greenaway, M. T., Vdovin, E. E., Ghazaryan, D., Misra, A., Mishchenko, A., Cao, Y., Wang, Z., Wallbank, J. R., Holwill, M., Khanin, Y. N., Morozov, S. V., Watanabe, K., Taniguchi, T., Makarovsky, O., Fromhold, T. M., Patanè, A., Geim, A. K., Fal'ko, V. I., Novoselov, K. S. & Eaves, L. Tunnel spectroscopy of localised electronic states in hexagonal boron nitride. *Communications Physics* **1**, 94, doi:10.1038/s42005-018-0097-1 (2018).
5 Cançado, L., Takai, K., Enoki, T., Endo, M., Kim, Y., Mizusaki, H., Jorio, A., Coelho, L., Magalhaes-Paniago, R. & Pimenta, M. General equation for the determination of the crystallite size L a of nanographite by Raman spectroscopy. *Applied Physics Letters* **88**, 163106 (2006).
